# High Throughput Screening Method for Identifying Potential Agonists and Antagonists of *Arabidopsis thaliana* Cytokinin Receptor CRE1/AHK4

**DOI:** 10.3389/fpls.2017.00947

**Published:** 2017-06-08

**Authors:** Pavel Klimeš, Dušan Turek, Pavel Mazura, Lucia Gallová, Lukáš Spíchal, Břetislav Brzobohatý

**Affiliations:** ^1^Laboratory of Plant Molecular Biology, Institute of Biophysics AS CR v.v.i. and Central European Institute of Technology, Mendel University in BrnoBrno, Czechia; ^2^Department of Chemical Biology and Genetics, Centre of the Region Haná for Biotechnological and Agricultural Research, Faculty of Science, Palacký University, OlomoucOlomouc, Czechia

**Keywords:** high throughput screening, plant growth regulators, cytokinin, CRE1/AHK4 receptor, laboratory automation

## Abstract

The CRE1/AHK4 cytokinin receptor is an important component of plants’ hormone signaling systems, and compounds that can alter its activity have potential utility for studying the receptor’s functions and/or developing new plant growth regulators. A high throughput method was developed for screening compounds with agonist or antagonist properties toward the CRE1/AHK4 cytokinin receptor in a single experiment using the Nanodrop II liquid handling system and 384-well plates. Potential ligands are screened directly, using a reporter system in which receptor signaling activity triggers expression of β-galactosidase in *Escherichia coli*. This enzyme generates a fluorescent product from a non-fluorescent substrate, allowing the agonistic/antagonistic behavior of tested compounds to be assayed in relation to that of an internal standard (here the natural ligand, *trans*-zeatin). The method includes a robust control procedure to determine false positive or false negative effects of the tested compounds arising from their fluorescent or fluorescent-quenching properties. The presented method enables robust, automated screening of large libraries of compounds for ability to activate or inhibit the *Arabidopsis thaliana* cytokinin receptor CRE1/AHK4.

## Introduction

Plant growth regulators are diverse groups of chemical messengers, including cytokinins, that affect various processes in plant growth and development. Compounds that specifically interact with plant hormone pathways have high utility in research and potential agronomic applications for improving crop yields or other aspects of crops’ performance (Basra and Lovatt, 2016; Koprna et al., 2016).

Currently there are several methods for testing substances’ effects on plants. The oldest and most straightforward is to apply them to whole plants in different developmental stages, parts of plants or plant cell cultures, then monitor the physiological, morphological, anatomic, and/or biochemical responses. However, these classical methods are not usually suitable for high throughput screening (HTS) due to the laboriousness and costs of both preparing the test materials (e.g., whole plants) and evaluating phenotypic effects of the treatments. Furthermore, although such techniques may identify substances with general effects on plant growth and development (for example, see Rodriguez-Furlán et al., 2016), they provide little or no information on the molecular level mechanisms involved.

A potentially more convenient and informative approach is to use an assay based directly on interactions between the tested compounds and proteins of interest (e.g., a cytokinin receptor, or reporter acting downstream of a targeted receptor) in a less complex system. Notably, heterologous systems have been used to express cytokinin receptors in a manner that functionally complements a two-component signaling pathway in a genetically modified yeast (Suzuki et al., 2001; Ueguchi et al., 2001) or *Escherichia coli* (Yamada et al., 2001). These systems can provide precise information about receptors’ signal transduction activities and ligand specificities. Moreover, transformed yeast expressing the CRE1/AHK4 receptor has been successfully used to screen for compounds with antagonistic activity (Arata et al., 2010), based on differences in the yeast’s growth (measured as changes in optical density at 600 nm, OD_600_) in 96-well plates. However, the method has several disadvantages for use in HTS applications, including complications associated with the yeast’s growth requirements and monitoring changes in the optical density.

The methodology reported in this work overcomes these disadvantages by using a strain of *E. coli* expressing a CRE1/AHK4 cytokinin receptor (Spíchal et al., 2004). CRE1/AHK4 signaling triggers expression of a β-galactosidase reporter gene, which can be detected by highly sensitive fluorescence measurements suitable for HTS. The described method provides a novel approach for screening cytokinin receptor agonists and antagonists in a single experiment, thereby identifying interesting compounds for further research and potential agronomical applications.

## Materials and Methods

### *E. coli* Strain and Plasmid

*Escherichia coli* strain KMI001 (*ΔrcsC, cps::lacZ*, Takeda et al., 2001) harboring the plasmid pIN-III – AHK4 was used in all the experiments reported here. The Arabidopsis cDNA that encodes the entire amino acid sequence of CRE1/AHK4 (UNIPROT ID: Q9C5U0) is cloned downstream of the lpp-lac promoter, followed by a prokaryotic ribosome-binding site, on pIN-III (between the unique *Bam*HI and *Sal*I sites) as described by Suzuki et al. (2001). Entire size of the construct is 10.7 kb.

### Materials and Reagents

Sodium carbonate, citric acid, disodium phosphate, magnesium sulfate, and dimethyl sulfoxide were purchased from PENTA s.r.o. (CZ). 4-methylumbelliferyl-β-D-galactopyranoside (4-MUGal), 4-methylumbelliferon (4-MU), 7-amino-4-methylcoumarin (7-AMC) and rifampicin were purchased from Sigma-Aldrich. A library of 93 newly synthesized compounds and ZOGA-090 was obtained from Palacký University Olomouc (CZ) for testing the screening setup. Chemical structure of ZOGA-090 is shown in **Supplementary Figure S1**. Chemical structures of the ligands were not disclosed due to intellectual property protection. Bacto^TM^ Casamino Acids were purchased from Becton, Dickinson and Company (United States). *Trans*-zeatin (tZ) was purchased from OlChemIm (CZ), and lysogeny broth (LB) medium from Duchefa Biochemie (NL).

Minimal (M9-505) medium, consisting of ZYM-505 medium (Studier, 2005) without N-Z-amine AS and yeast extract, was prepared and enriched with 0.1% v/v Casamino acids. The final concentration of ampicillin in this medium was 50 μg/ml and final concentration of the solvent (DMSO) was ∼1% (v/v). Concentration of DMSO up to 3% did not significantly change response of the detection culture. Radioactive-labeled [^3^H]tZ (1,3 TBq/mmol) was obtained from the Isotope Laboratory, Institute of Experimental Botany, AS CR, Prague (CZ) scintillation cocktail was purchased from Perkin Elmer (United States).

### Live-Cell Cytokinin-Binding Assay

Intact *E. coli* cultures (strain KMI001), expressing CRE1/AHK4 cytokinin receptor (Suzuki et al., 2001; Yamada et al., 2001), were grown at 25°C overnight. M9 liquid medium, supplemented with casamino acids [0.1% (w/v)] and ampicillin (100 μg/ml), were used to reach OD_600_ ∼1–1,4. The assay described by Romanov et al. (2005) was performed with slight modifications.

Each sample contained 1 ml of the overnight cell culture, 3 pmol of [^3^H]tZ and various concentrations of unlabeled tZ/other tested compound (0.1 nM–50 μM). Negative control contained 3 pmol of [^3^H]tZ and 0.1% (v/v) dimethylsulfoxide (DMSO; solvent), instead of the unlabeled compound. After 30 min incubation at 4°C, the sample was centrifuged (8,000 rpm, 4 min, 4°C) and supernatant was removed. Bacterial pellet was resuspended in 50 μl dH_2_O. Subsequently, 1 ml of scintillation cocktail was added. Radioactivity was measured by a Hidex 300 SL scintillation counter Hidex (FL). High excess of unlabeled tZ (at least 3000-fold) was used for competition, to discriminate between specific and non-specific binding.

### HTS Equipment

A Nanodrop II liquid handling system (BioNex Solutions, San Jose, CA, United States), was used for all pipetting steps. BioNex Nanodrop II accessories can be mounted on two nests, mostly used for microtitration plates. There are also two positions for trays (containing in this case *E. coli* suspension and decontaminating bleach solution) or PCR tube holders. *E. coli* was cultivated using a microplate shaker with a controlled heating platform (ThermoMixer C, Eppendorf) and heated lid (ThermoTop, Eppendorf). For screening, sterile transparent 384-well plates (Corning, United States) were used. Optical densities (OD_600_) and fluorescence intensities of the β-galactosidase-catalyzed reaction product (excitation and emission maxima: 365 and 448 nm, respectively) were measured using an Infinite M1000Pro plate reader (Tecan, CH). In case the HTS automation is not available the method could be downscaled and adapted for manual pipetting similarly as described by Spíchal (2011).

### Statistical Analysis

For multiple comparison analysis of the acquired data sets *t*-tests were used. Mean responses to test compounds, relative to DMSO controls, were assessed by calculating *P*-values. These were deemed significant if lower than α*_ADJ_*, the nominal significance level following Šidák correction, derived from α*_ADJ_* = (1-(1-α)ˆ1/m) = 0.00054, where α = 0.05 and m = 95 (Šidák, 1967). To describe the separation between responses to an internal standard (tZ at 50 nM) and both a positive control and a negative control (50 μM tZ and ZOGA-090, respectively), the Z′-factor described by Zhang et al. (1999) was used. All calculations were performed in MS Excel 2013.

## Results

### Preparation and Optimization of Use of the *E. coli* Detection Culture

#### General Description of the *E. coli* Detection Culture

**FIGURE 1:**
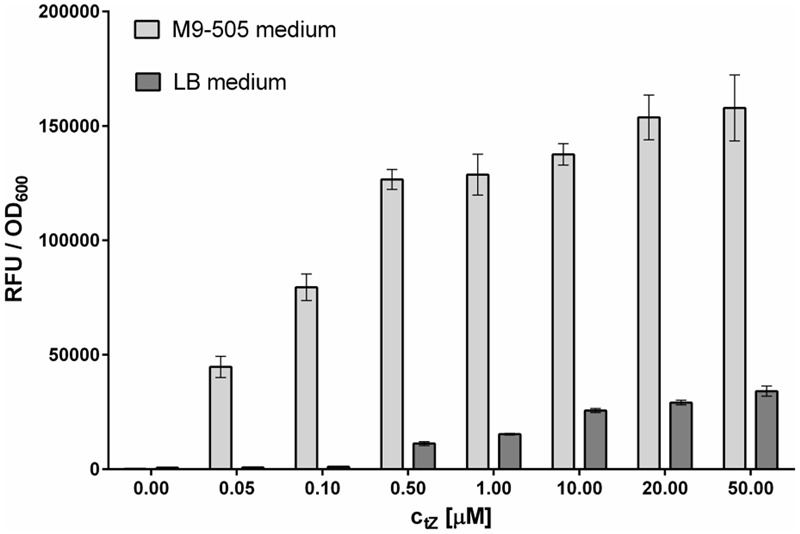
Comparison of sensitivity of *Escherichia coli* reporter cultures grown on LB and M9-505 media. Experiments were performed in 96-well format involving incubation with tZ for 5 h at 25°C and 800 rpm shaking, followed by 30 min incubation with 4-MUGal substrate at 37°C and 800 rpm shaking, and finally measurement of fluorescence from mixtures of 50 μl of *E. coli* suspension and 100 μl of 0.2 M sodium carbonate. Means ± SD (*n* = 4) of ratios of final RFU to OD_600_ shown. Cultures grown on M9-505 medium clearly provide much higher sensitivity than cultures grown on LB medium.

As described by Spíchal et al. (2004), *E. coli* strain KMI001 expressing the CRE1/AHK4 cytokinin receptor has been used to develop a system for studying the receptor’s interaction with potential agonists/antagonists. In this system, the CRE1/AHK4 receptor (a kinase) generates signal after interacting with an activating ligand presented in the growth medium. Further signal transduction triggers an engineered operon leading to expression of the reporter enzyme β-D-galactosidase (Suzuki et al., 2001), at a level related to the ligand’s concentration, activating properties and duration of interaction with the receptor (Spíchal et al., 2004), up to a saturation level, beyond which increases in ligand concentration only result in marginal increases in signaling intensity (**Figure 1**). The assay results are expressed in terms of optical density of the bacterial culture and fluorescence intensity of the β-galactosidase-catalyzed reaction product, and the strength of the ligand-receptor interaction is described as the ratio between fluorescence intensity and optical density. In the study reported here, limitations of the detection system and optimal conditions were experimentally investigated.

#### Selection of Growth Medium

**FIGURE 2:**
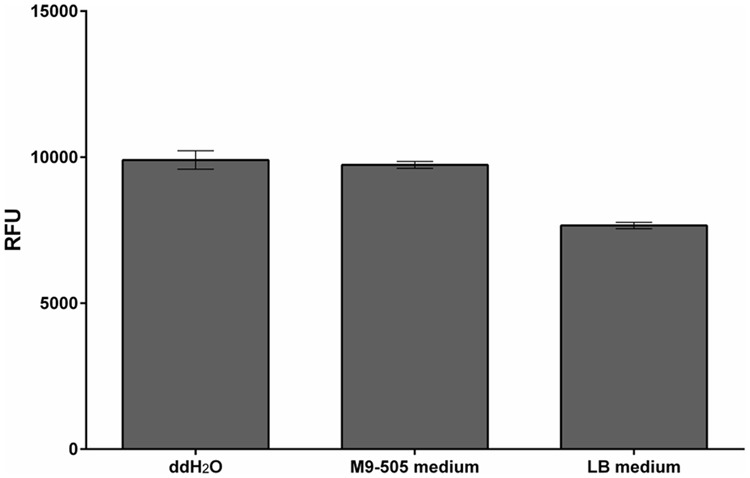
Quenching properties of M9-505 and LB media. Comparison of quenching of fluorescence of the enzymatic reaction product (4-MU) by M9-505 and LB media. Means ± SD (*n* = 3) obtained in tests with solutions consisting of 30 μl of the media (or water), 9 μl of 1.2 M sodium carbonate and 1 μl of 2 mM 4-MU contained in wells of a 384-well plate. LB medium clearly quenched the fluorescence substantially more than M9-505 medium.

The composition of the culture medium profoundly affects the *E. coli* strain’s performance. Here, its performance was compared in tests with tZ and two types of media – Lysogeny broth (LB) medium and M9-505 minimal medium (modified ZYM-505 medium, Studier, 2005). LB medium is commonly used because it is simple to prepare and allows stable growth of bacteria, while minimal medium is used in situations where the medium’s composition must be fully defined. As expected, *E. coli* grew faster in LB medium than in M9-505 medium, but although the cultures reached higher optical densities in LB medium, the measured fluorescence was higher in M9-505 medium, as shown by the final relative fluorescence unit (RFU)/OD_600_ ratios (**Figure 1**). Thus, the M9-505 medium affords higher sensitivity. Moreover, further tests showed that the LB medium quenches the fluorescence signal by ∼20% (**Figure 2**). Thus, M9-505 medium was used in all further experiments.

#### Effects of the Pre-cultivation Procedure on the Detection Culture’s Sensitivity

Fresh KMI001-AHK4 detection culture must be prepared for each experiment. In the standard previously reported procedure a colony picked from a Petri dish or a portion of frozen stock of *E. coli* strain KMI001-AHK4 is used to start a 1 ml overnight culture, which is inoculated the following day into fresh medium to prepare the detection culture used to test compounds’ interaction with the receptor (Mizuno and Yamashino, 2010; Spíchal, 2011). However, this straightforward procedure provides inconsistent responses to a standardized concentration of tZ due to variations in duration of cultivation of the *E. coli* (from the same frozen stock), and both the volume and dilution of the overnight culture (**Supplementary Figure S2**).

**FIGURE 3:**
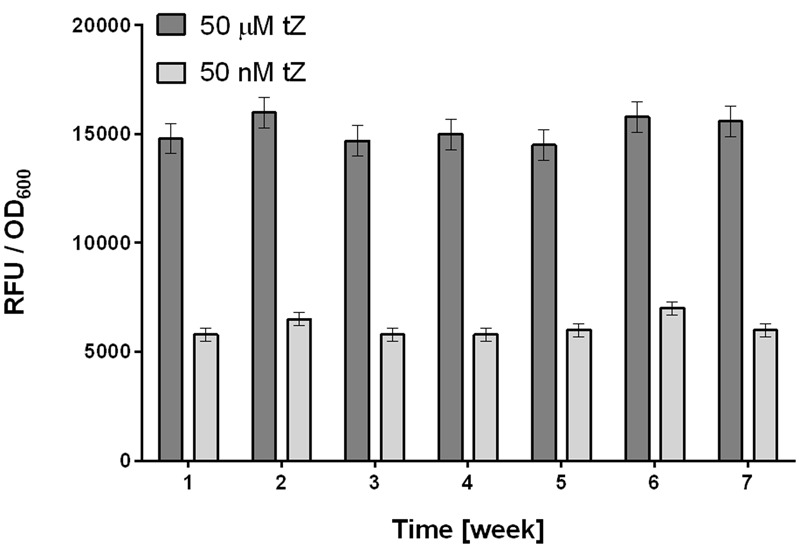
Stability of responses to tZ of the optimally prepared *E. coli* KMI001-AHK4 detection culture. Frozen stocks of detection culture were tested weekly for 7 weeks, and provided stable responses to tZ at both low (50 nM) and high (50 μM) concentrations. Stocks of detection culture were prepared as follows. 50 ml of M9-505 medium in a 250 ml Erlenmeyer flask was inoculated with a stock KMI001-AHK4 culture then incubated at 25°C, with 250 rpm shaking for 20 h. The *E. coli* was then harvested by centrifugation (10,000 *g* for 10 min) and resuspended in 1 ml of 50% glycerol and M9-505 medium (1:1). Portions (50 μl) of this mixture were frozen in liquid nitrogen and stored at –80°C, then resuspended in 40 ml of M9-505 medium immediately before use as a detection culture in tZ response tests involving fluorescence measurement as in the standard high throughput screening (HTS) protocol (see below). Bars in the graph represent means ± SD (*n* = 4).

To avoid this variability, we developed the following procedure. First, a larger volume (50 ml) of overnight culture is prepared from a stock of frozen culture with verified activity in tests with tZ. The next day the overnight culture is harvested, then prepared portions are frozen in liquid nitrogen, and stored at -80°C. These portions are directly diluted in medium and ready for use in screening. The procedure has several important advantages. The step that generates most variability in properties of the detection culture (cultivation of an overnight culture), is omitted. Frozen stocks are immediately available for HTS screening, experiments can be started at any time, and they provide highly stable responses to tZ. These responses are not linear but provide (in our experimental setup) a measurement window between 50 nM and 50 μM, which sacrifices resolution at the upper end of the concentration scale, but enables reliable detection of low concentrations of tZ, which is crucial for detecting antagonists by the HTS method (**Figure 3**).

#### Effect of Detection Culture Cultivation Temperature on CRE1/AHK4 Signaling

**FIGURE 4:**
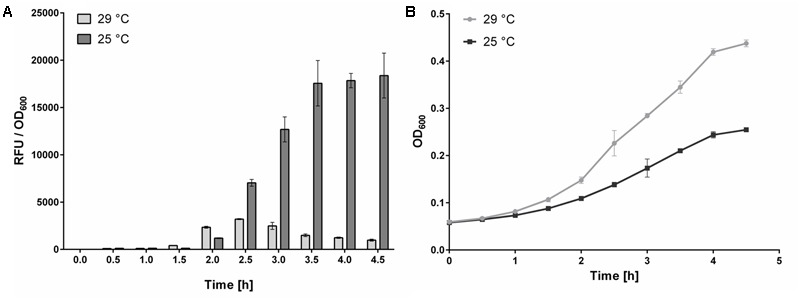
Effect of temperature on AHK4 signaling. Signal to bacterial density (RFU to OD_600_) ratios, and bacterial densities, obtained following incubation (for indicated times) of *E. coli* KMI001-AHK4 at 25 and 29°C in M9-505 medium supplemented with 1 μM tZ. 200 μl portions of KMI001-AHK4 detection culture were dispensed into two 96-well plates with tZ. One was cultivated at 25°C, and the other at 29°C, for 5 h with shaking at 800 rpm in a ThermoMixer C (Eppendorf). Every 30 min. three 30 μl samples were taken from both sets of *E. coli* suspensions, their optical density was measured then 0.3 μl of 50 mM 4-MUGal was added and incubation continued in a 384-well plate at 37°C with shaking at 1,400 rpm for 30 min. Then the reaction was stopped by adding 9 μl of 1.2 M sodium carbonate and fluorescence was measured. **(A)** The RFU/OD_600_ ratios clearly show that detection culture cultivated at 29°C was much less sensitive than otherwise identically cultivated culture at 25°C. **(B)** Growth curves of detection cultures follow the classical trend, but in contrast to expectations the higher densities of *E. coli* cultivated at 29°C are associated with substantially lower RFU/OD_600_ ratios. Thus, the KMI001-AHK4 detection culture must be incubated with test compounds at a constant temperature to obtain reproducible responses. In both **(A,B)**, data shown are means ± SD (*n* ≥ 3).

The first phase of the assay involves incubating portions of the *E. coli* KMI001-AHK4 detection culture with test compounds for several hours at 25°C (Mizuno and Yamashino, 2010; Spíchal, 2011). Growth rates of *E. coli* are influenced by available oxygen levels in wells of the 384-well plates used (Duetz et al., 2000). Thus, to ensure sufficient aeration of 30 μl suspensions of bacteria growing in the M9-505 medium in the plates a high-speed microplate shaker (MixMate, Eppendorf, Germany) was initially used. However, results of experiments with this shaker had poor reproducibility, possibly due to transfer of excess heat from the shaker into the 384-well plate, which increased the cultivation temperature up to 29°C and thus apparently affected the signaling pathway. To confirm this hypothesis, further experiments were performed with incubation at both 25 and 29°C, using plates shaken in microplate shakers with a controlled heating platform (ThermoMixer C, Eppendorf) and heated lid (ThermoTop, Eppendorf). The results show that cultivation at 29°C reduces CRE1/AHK4 signaling activity in the *E. coli* KMI001-AHK4 detection system by 95% relative to the activity at 25°C (**Figure 4A**), although the bacteria grew faster (**Figure 4B**). Thus, the better growth conditions clearly suppress the signaling pathway or expression of the reporter enzyme, even in the presence of the strong agonist tZ (1 μM). Moreover, during the (5-h) incubation phase water evaporated from medium condensed on the inner side of the plate cover. However, using the heated lid in experiments stops water condensation and ensures thermal homogeneity across the plate. This setup also avoids the need for methods such as placing a wet paper towel under the plate in the plate holder to compensate for evaporation (Zhao, 2003).

The results as well as published data (Mizuno and Yamashino, 2010; Spíchal, 2011) clearly show that the receptor signaling pathway is suppressed by the higher temperature during the incubation of compounds with detection culture, thus it is highly important to keep the temperature constant (preferably at 25°C, unless further analyses indicate that another temperature is closer to optimal). They also show that the temperature can be robustly controlled using a microplate shaker that can control the temperature of both the shaking platform and lid.

### Properties of Compounds that Interfere with the HTS

#### Interference by Compounds’ Optical Properties with the Optical Density Measurements

After mixing *E. coli* detection culture with test compounds, optical densities were measured at 600 nm. Clearly the OD_600_ will be increased, at least initially, by any compound that absorbs light at 600 nm, and such changes in OD_600_ must be considered at the end of the experiment. This is not straightforward because compounds may be metabolized or degraded during the incubation. However, significant differences in OD_600_ of detection cultures incubated with test compounds, relative to those of controls incubated with DMSO, that persist throughout the incubation must be accounted for when evaluating the data and identifying potential agonists/antagonists.

#### Interference of Fluorescent Compounds, Fluorescence Quenchers, or Their Precursors, with Fluorescence Measurements

**FIGURE 5:**
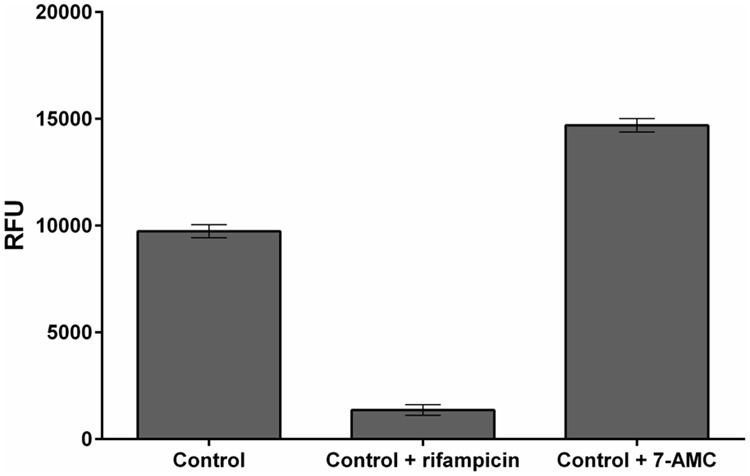
Example of fluorescent analog and fluorescence-quencher. Effects of rifampicin and 7-AMC in M9-505 medium on fluorescence from 4-MU (Control), in wells of a 384-well plate. Final control solutions contained 30 μl medium (M9-505), 9 μl of 1.2 M sodium carbonate, and 1 μl of 0.2 mM 4-MU. 7-AMC and rifampicin were added to solutions (at a final concentration of 50 μM), as a fluorescent analog of 4-MU and a fluorescence-quencher, respectively, under the same conditions as test compounds.

When large libraries of compounds are screened in HTS programs some could have similar fluorescent properties to 4-MU (measured at the end of experiment), or quench fluorescence from the formed 4-MU, thereby interfering with its determination. In addition, degradation or metabolization of compounds during their incubation with the *E. coli* detection culture could affect their fluorescent properties. To assess such potential effects (which clearly must be distinguished and accounted for) we investigated the interference caused by the model fluorescent compound 7-amino-4-methylcoumarin (7-AMC) and fluorescence-quenching compound rifampicin (Richter et al., 2015; Żamojć et al., 2015). As anticipated, these compounds respectively increased and decreased fluorescence recorded in the assay (**Figure 5**).

The results show that fluorescent or fluorescence-quenching compounds may substantially affect results of the screening procedure, so we recommend inclusion of a reference plate to identify such compounds in the compound library.

#### Effects of Compounds on Growth of *E. coli*

After incubating test compounds with *E. coli* detection culture for 5 h the optical densities of all samples in the 384-well plate are measured. In the presence of a strong agonist activation of the signaling pathway reduces growth of the *E. coli* due to intense expression of the reporter enzyme, as shown by differences (of about 20%) in the optical densities of negative and positive controls (containing DMSO and tZ, respectively). This significant decrease in optical density can be used as a secondary marker (in addition to high fluorescence) for identifying agonists. False positive agonists usually lack this property, as inhibitors of the signaling pathway generally do not perturb growth of the *E. coli*, although the theoretical possibility cannot be excluded. Some screened compounds may also potentially inhibit or promote *E. coli* growth. The possibility that a few test compounds may have complex effects that complicate detection of effects on the CRE1/AHK4 receptor *per se* is a limitation of the described HTS method.

### HTS Method

#### Internal Standard

In order to distinguish between agonists, antagonists, and compounds that do not interact with the receptor in a single experiment, we introduced an internal standard procedure by adding the natural ligand tZ to the growth medium (to a final concentration of 50 nM) at the start of the experiment. At this concentration, tZ triggers a predetermined weak response of the receptor signaling pathway. In this setup agonists are able to trigger signaling pathway even to higher level leading into increase of the fluorescence intensity at the end of the experiment. Competitive antagonists compete with tZ for the same binding site of the receptor and thereby decrease fluorescence output. In the case of uncompetitive antagonism the decrease in the fluorescence output is also recorded. Compounds that do not interact with the receptor have no effect on fluorescence.

#### Reference and Screening Plate Setup

As compounds tested in a HTS may have properties that interfere with fluorescence from 4-MU (**Figure 5**), two 384-well plates (screening and reference) are used in parallel to investigate the screened compounds’ fluorescence properties and preclude artifacts.

Wells of the screening plate are filled with test compound solutions (concentration during test 50 μM), mixtures containing the *E. coli* KMI001-AHK4 detection culture and 50 nM of tZ then incubated for 5 h with shaking at 1,400 rpm. After this the substrate (4-MUGal) is added and the resulting mixtures are incubated for another 30 min at 37°C. Finally, the enzymatic reaction is stopped by adding 1.2 M sodium carbonate solution and fluorescence of the formed 4-MU is measured.

**FIGURE 6:**
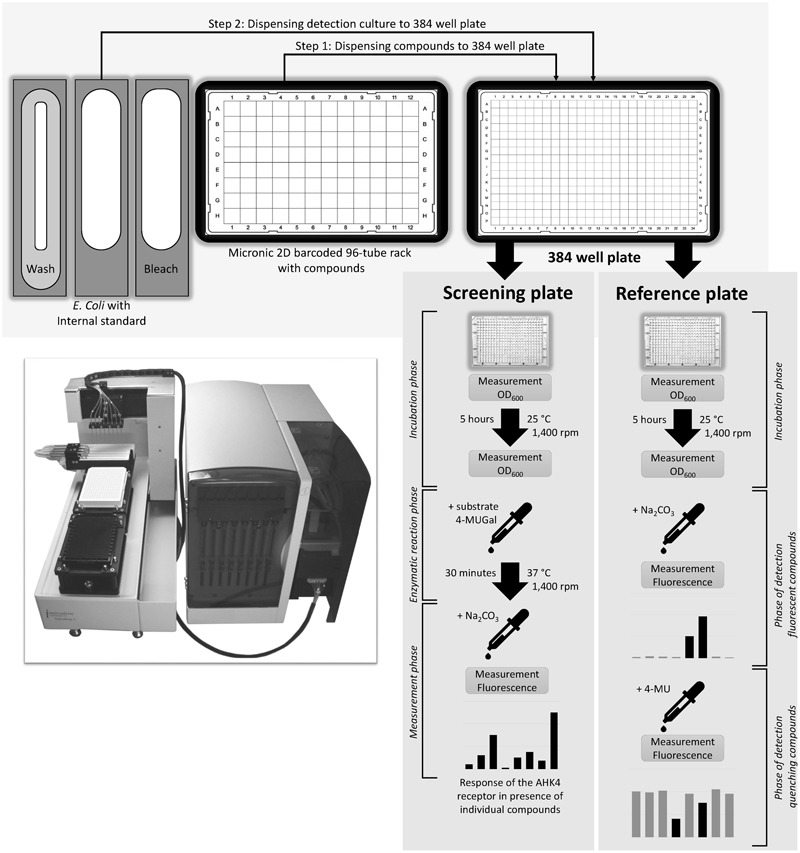
High throughput screening scheme. A liquid handling system is used for pipetting compounds, *E. coli* detection culture, 4-MUG substrate, stop solution (Na_2_CO_3_) and fluorescence standard (4-MU). The screening plate is used for direct investigation of test compounds’ effects on the AHK4 receptor (agonistic, antagonistic, or neutral). The reference plate is used for determining properties of test compounds that could interfere with the fluorescence measurements (by fluorescing like 4-MU or quenching fluorescence).

The reference 384-well plate is treated identically, except that addition of the 4-MUGal substrate and incubation at 37°C are omitted, and the fluorescence measurements (after addition of sodium carbonate) identify compounds that may fluoresce like 4-MU, by yielding stronger fluorescence than controls containing DMSO. A second fluorescence measurement is taken after adding 300 nl of 6 mM 4-MU to each well, to identify compounds that significantly reduce fluorescence relative to DMSO controls and thus are fluorescence quenchers. At this point the fluorescence resulting from incubations with some test compounds may also exceed control levels, indicating non-specific additive interaction between 4-MU and those compounds. Thus, in all cases fluorescence properties of the hits must be evaluated by comparing results obtained from the screening and reference plates, as schematically illustrated in (**Figure 6**).

#### Statistical Evaluation of the Screening Results and Empirical Rules for Hit Sorting

In the screening reported here each test rack contained 93 compounds and three controls—a positive control (the natural ligand tZ), a negative control (the antagonist ZOGA-090; Gallová et al., 2016) and a neutral control (DMSO, for quantifying the response solely to the internal standard)—in four technical replicates. The fluorescence to optical density ratio obtained in each case was calculated to normalize the results with respect to growth of the detection culture. The screening hits were defined as ligands that interact with the CRE1/AHK4 receptor and significantly increase or decrease the response induced by the internal standard. The means of the measured responses to tested compounds and the DMSO control were compared using Student’s *t*-test. Due to the high number (95) of independent tests, the α (nominal significance) level were subjected to Šidák adjustment (α_ADJ_ = 0.00054) to avoid type I errors (statistically significant but false positive test results; Abdi, 2007). Thus, statistically significant results (hits) must be checked to exclude possible false positive or negative effects caused by the compounds’ non-specific interference with either fluorescence/absorbance measurements or effects on *E. coli* growth. Due to a high discovery rate of hits, to facilitate distinction of those warranting the closest further attention we applied empirical rules to sort identified hits into five groups: strong agonists, agonists, neutral agents (which do not induce a significantly different response from DMSO controls), antagonists and strong antagonists. Strong agonists were defined as compounds that induced responses at least mid-way between those of tZ at the test and internal standard levels (50 μM and 50 nM, respectively). The strong antagonists were defined as compounds inducing responses weaker than midway between responses to the internal standard (plus DMSO) and ZOGA-090. The agonists and antagonists were defined as compounds that induced responses that were weaker than these hits defining level, but significantly different from those induced by the internal standard.

#### Hit Validation

The identification of HTS hits is based on the activity of the *lacZ* reporter down-stream of the heterologous signaling cascade, thus, it can’t be excluded that the hit molecule influences the reporter activity by mechanism different from the interaction with the receptor active site, e.g., through interaction with other components of the cascade. For this reason a validation step is included into the screening procedure. The validation is based on the analysis of the affinity of the hit to the receptor ligand-binding site. Compounds selected by HTS screen as potential agonists and/or antagonists of CRE1/AHK4 were tested for their ability to compete with tritium labeled natural ligand [^3^H]tZ for binding into the receptor active site using the live-cell cytokinin-binding assay published by Romanov et al. (2005). The validation step shows whether the tested compound directly interacts with the receptor active site and unambitiously confirms the mode of action of the HTS selected hit. In positive case the hit is classified as a confirmed specific agonist or antagonist of the receptor CRE1/AHK4.

### HTS Workflow

**FIGURE 7:**
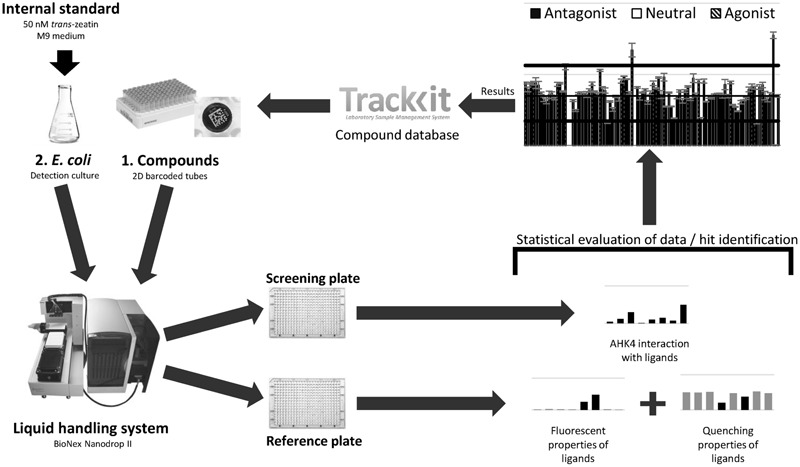
High throughput screening workflow scheme. This is a complex procedure that involves selecting compounds for screening from the database, preparation of samples in 2D barcoded tubes, the screening procedure using the liquid handling system, statistical evaluation of obtained data and finally uploading of the results into the database.

The starting point of a HTS program is selection of compounds from a suitable database (here in TrackIt software). Selected compounds are prepared in the standard format, with 93 new compounds (one full rack with three positions for controls) diluted in DMSO at a stock concentration of 5 mM. Sample tubes are barcoded to enable tracking of compounds in the screening and selection of tubes for further analysis of hits. The results obtained for the screened compounds are evaluated and stored, together with procedure details, in the database for later processing, thereby closing a cycle (**Figure 7**).

### Example of HTS Performance with a Full Set of 93 Compounds

#### Preparation of Bacterial Detection Culture

Fifty μl of frozen (-80°C) *E. coli* KMI001-AHK4 bacterial detection culture was thawed on ice (10 min) and then diluted with 40 ml of M9-505 medium. The resulting suspension was supplemented with the internal standard (2 μl of 1 mM tZ; final concentration, 50 nM).

#### Automatic Liquid Handling Procedure

All pipetting steps were performed by a Nanodrop II liquid handling system (Bionex, NL). Each test compound and each of the control (0.3 μl) was dispensed from a tube rack (Micronic, NL) into 4 wells of a 384-well transparent microtitration screening plate (Corning, United States), then 30 μl of detection culture was dispensed into each well of the plate (Supplementary Data Sheet 1). The procedure is captured on video^1^.

#### Incubation and Detection Phases

The screening plate was incubated at 25°C with shaking at 1,400 rpm for 5 h (ThermoMixer C, Eppendorf). The optical density (OD_600_) of the bacterial culture was then measured using an Infinite M1000Pro plate reader (Tecan, CH). 0.5 μl of 50 mM of the β-galactosidase substrate (4-MUGal in DMSO, final concentration 0.80 mM) was added to all wells and the plate was incubated at 37°C with shaking at 1,400 rpm for 30 min in a ThermoMixer C instrument (Eppendorf). The 4-MUGal hydrolysis catalyzed by the β-galactosidase reporter enzyme was stopped by adding 9 μl of sodium carbonate (1.2 M). The fluorescence resulting from incubation with each test compound was measured using an Infinite M1000Pro plate reader (Tecan), with excitation at 365 nm and emission at 448 nm, then expressed in terms of a RFU/OD_600_ ratio.

A reference plate with wells filled with compounds and *E. coli* was also incubated for 5 h, as above. Then 9 μl of the sodium carbonate solution was dispensed to each well, fluorescence was measured and fluorescent compounds were detected. Finally, 300 nl of 6 mM 4-MU was dispensed to each well and fluorescence was measured again to detect compounds that quench the fluorescence.

#### Results of Test Run with a Full Set of 93 Compounds

In our setup a test set of 93 compounds is optimal because it completely fills a 96-well source plate (together with three controls). Thus, if large numbers of compounds are to be screened they should ideally be divided into groups of 93. Therefore, to assess the screening method’s performance we subjected 93 compounds for which no information regarding activity was available, and subjected them to the screening procedure described above.

The wells in the 384-well screening plate were occupied by quadruplicate mixtures containing the 93 unknown compounds, at 372 positions in total, and three controls (tZ as a natural agonist of the receptor, ZOGA-090 as a known antagonist and DMSO to quantify the response to the internal standard) at 12 positions in total.

**FIGURE 8:**
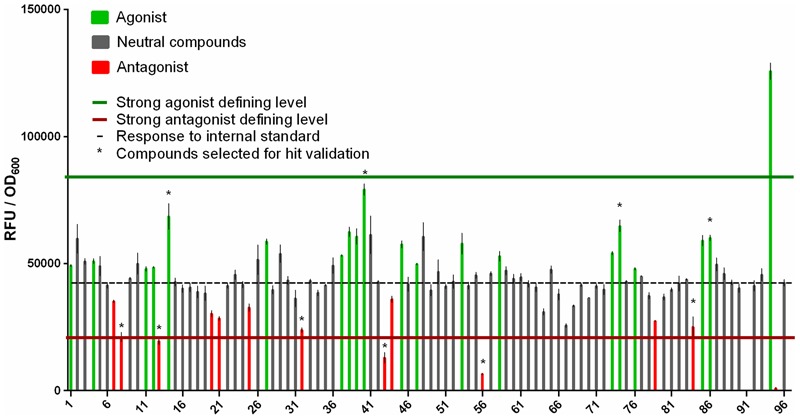
Results of the test screen of 93 unknown compounds for ligands that interact agonistically or antagonistically with the CRE1/AHK4 receptor. Comparison of responses (RFU/OD_600_ ratios) induced by each compound and the internal standard (no. 96, dashed line). The upper horizontal (green) line indicates the hit defining level for strong agonists (inducing stronger responses than the midpoint between responses induced by the internal standard and 50 μM tZ [no. 94]), while the lower horizontal (red) line indicates the hit defining level for strong antagonists (inducing weaker responses than the midpoint between responses induced by the internal standard and 50 μM ZOGA-090 [no. 95]). Ligand 91 suppresses growth of the detection culture, and thus yields no fluorescence. ^∗^Compounds selected for hit validation.

The responses induced by the internal standard, natural agonist tZ and antagonist ZOGA-090, expressed as RFU/OD_600_ ratios, were 42,427 ± 1,292, 125,728 ± 3,235, and 898 ± 143, respectively. In addition, the separations between the response to the internal standard and both the positive control (tZ) and negative control (ZOGA-090) were very good, with Z′-factors of 0.84 and 0.90, respectively. Moreover 19 agonists and 12 antagonists (three defined as strong antagonists) were identified among the 93 unknown compounds. One compound suppressed growth of the detection culture, so its effect on the CRE1/AHK4 receptor could not be determined using our reporter system (**Figure 8**). None of the compounds significantly influenced the fluorescence measurement (**Supplementary Figures S3, S4**).

**FIGURE 9:**
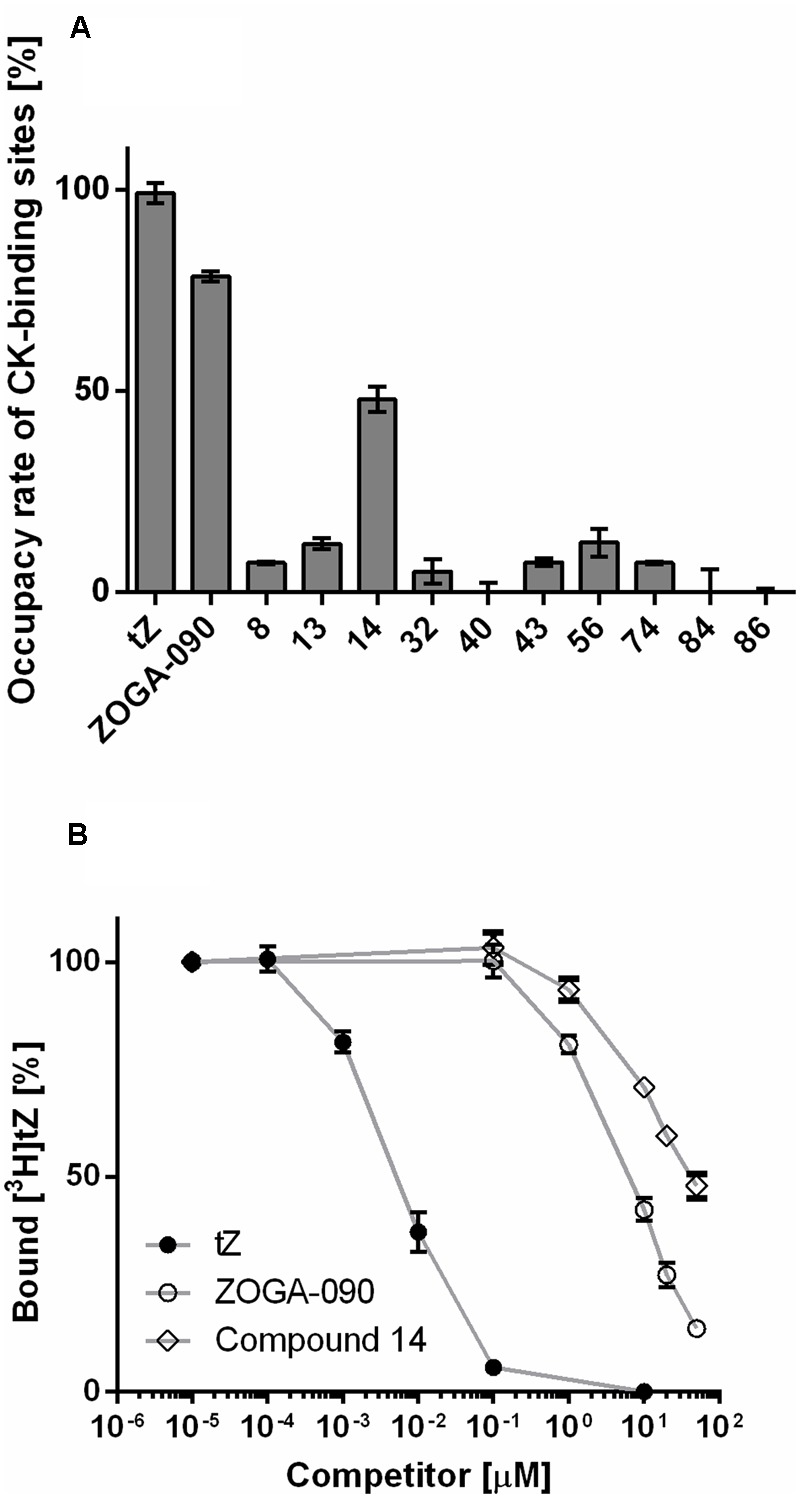
Hit validation. **(A)** Live-cell cytokinin-binding assay with CRE1/AHK4 cytokinin receptor. Tested compounds (in 20 μM concentration) competed with radioactive-labeled [^3^H]tZ at the receptor active site. Maximum binding affinity [100%] is related to 10 μM tZ. **(B)** The detailed analyses confirmed that compound 14 competes for binding into the CRE1/AHK4 cytokinin binding site in the dose dependent manner although the binding is weaker than for ZOGA-090.

Ten compounds selected by HTS screen as potential agonists and/or antagonists of CRE1/AHK4 were tested for their ability to compete with tritium labeled natural ligand [^3^H]tZ for binding into the CRE1/AHK4 active site. The excess of the tested compounds (concentration > 6,500-times higher than [^3^H]tZ) was used to select the compounds capable of the effective ligand displacement. Cytokinin tZ (10 μM) and cytokinin antagonist ZOGA-090 (20 μM) were used as the agonist and antagonist positive controls, respectively. From the 10 HTS-selected hits one potential agonist (compound 14) showed effective displacement of the natural ligand from CRE1/AHK4 active site (**Figure 9A**). The detailed analyses confirmed that compound 14 competes for binding into the CRE1/AHK4 cytokinin binding site in the dose dependent manner (**Figure 9B**).

## Discussion

The optimization of the screening method described here shows that M9-505 medium rather than rich LB medium should be used to cultivate the detection culture as it provides high fluorescence intensity at the end of the experiment (and thus higher sensitivity). In addition, the test compounds should be incubated with the detection culture at a carefully controlled temperature at 25°C because the higher temperature suppresses activation of the signaling pathway. Use of a Thermomixer C instrument enables the required control via its platform and lid heating systems. The biological variability in *E. coli* cultures cultivated overnight could cause significant problems, but they can be avoided by preparing frozen stocks of *E. coli* KMI001-AHK4 culture with verified activity for use in the screening procedure.

The screening setup was designed to distinguish both agonists and antagonists in a single run by including a low concentration of the natural agonist tZ in each assay mixture. Thus, agonists and antagonists should respectively increase and decrease the response of the CRE1/AHK4 receptor to the natural agonist, provided false hits caused by compounds with fluorescent or fluorescence-quenching properties are distinguished using a reference plate.

Comparison of screening and reference plates enables identification of compounds with properties that interfere with fluorescence signals from 4-MU, thereby complicating evaluation of their effects on CRE1/AHK4 signaling. Thus, incorporation of this step eliminates false agonist/antagonist hits during the screening procedure and saves the work and time needed for reevaluation of false hits.

A suitable system for storing and tracking samples is vital for HTS. Thus, the potential ligands library was stored in Micronic 2D barcoded tube racks, thereby assigning a unique number and rack position to every compound. In addition, rack positions and screening results were uploaded into an online (TrackIt) database, thus facilitating searches for specific compound and maintaining direct links between the screening information and individual compounds.

The procedure for identifying hits in HTS depends on two sets of factors. One set consists of inherent features of the screening assay that influence the system’s technological and biological variability, and thus the ability to separate signal and noise. The other set consists of factors associated with hits’ significance, such as the screening objectives, planned follow-up analyses, and budgetary constraints.

The assay presented here exploits a signaling cascade in living *E. coli* cells, so inevitably there is a significant amount of inherent biological variability in the system. Nevertheless, the optimized assay behaves highly consistently, enabling easy discrimination of small differences between responses induced by test compounds. A major screening objective was to identify the best agonists/antagonists of the CRE1/AHK4 receptor, so we decided to define strong agonists and strong antagonists by comparing compounds’ responses with those of the well-defined agonist tZ and antagonist ZOGA-090 using robust statistical criteria. Use of such criteria for detecting important hits from a test library of compounds could be extended to hit selection in HTS more generally. HTS should select the potential agonists and/or antagonists of the receptor CRE1/AHK4, i.e., compounds with specific affinity to the receptor active site. However, the selection can contain also false positive hits influencing the assay read-out message through interaction with other members of the down-stream signaling cascade and/or the reporter activity. A validation step is thus needed to discriminate the compounds interacting with the receptor out of its active site, e.g., with kinase out-put domain, non-specific inhibitors of phosphotransfer, or inhibitors of β-galactosidase. In the presented method the validation step is based on the direct confirmation of the hit interaction with the ligand-binding site. However, whatever cytokinin-specific bioassay can be used for the further functional validation of a cytokinin receptor agonist or antagonist as described, e.g., by Doležal et al. (2006) and Spíchal et al. (2007), respectively.

The developed method can be used for screening libraries of compounds to find potential plant growth regulators that specifically target cytokinin perception. Moreover, its automation, miniaturization and ability to identify agonistic or antagonistic compounds in a single step enable highly efficient screening, and the extension of the method enables detection of false hits due to test compounds having either fluorescence or fluorescence-quenching properties. The method is capable to uncover interfering properties of screened ligands even when these properties are developed just during the screening procedure.

## Author Contributions

PK programmed the robot, DT optimized cultivation of *E. coli* cultures, PK and DT performed the experiments, and analyzed data, PM invented the method, designed the research and analyzed data, LG created compound library, designed and performed the validation step, LS designed the research and interpreted data, BB supervised all facets of the project. All authors contributed to write the manuscript.

## Conflict of Interest Statement

The authors declare that the research was conducted in the absence of any commercial or financial relationships that could be construed as a potential conflict of interest.

## Footnotes

1 https://youtu.be/EqW_bHA8Gd8

## Supplementary Material

FIGURE S1 ZOGA-090 (*N*-(3-methylbut-2-en-1-yl)-5*H*-pyrrolo[3,2-*d*]pyrimidin-4-amine) is a 9-deaza derivative of a natural cytokinin isopentenyladenine (iP). The lack of nitrogen in position 9 decreases affinity to the cytokinin receptors and mainly causes complete loss the receptor activation capacity.

FIGURE S2 A detection culture that provides a stable response is required for the HTS procedure. Responses to 100 nM tZ of detection cultures prepared from cultures grown overnight (from frozen stocks) for times varying from 15 h to 23 h cover a 2-fold range (S12 A). Volumes of detection cultures ranging from 1 to 4 ml prepared from overnight cultures (started at the same time) provide (at least in the illustrated case) linear responses to 100 nM tZ (S12 B). However, the dilution also affects properties of the detection culture, as illustrated by results obtained with 20-, 50- and 100-fold dilutions of overnight culture (S12 C). Thus, even after optimizing the duration of cultivation, cultivation volume and dilution of overnight culture, our efforts to prepare stable detection cultures from overnight cultures for every experiment were not entirely successful.

FIGURE S3 Fluorescence of compounds measured in a 384-well reference plate after 5 hours incubation with *E. coli* strain KMI001-AHK4 and addition of sodium carbonate solution. There were significant differences between fluorescence induced by several compounds and DMSO (in wells O23, O24, P23, P24), but these measured values did not affect the evaluation of the data set (the response induced by the internal standard was ∼6000 RFU).

FIGURE S4 Quenching of fluorescence of compounds measured in a 384-well reference plate after 5 hours incubation with *E. coli* strain KMI001-AHK4 and addition of sodium carbonate solution and 4-MU. The measured fluorescence intensities were not significantly different from those induced by control (DMSO) (No. 96) at the significance level α_ADJ_ = 0.00054 according to Šidák correction.
