# Supplementary figures and images for: High Throughput Screening Method for Identifying Potential Agonists and Antagonists of Arabidopsis thaliana Cytokinin Receptor CRE1/AHK4
Source: Front Plant Sci. 2017 Jun 8;8:947. doi: 10.3389/fpls.2017.00947 (PMC5463364; doi:10.3389/fpls.2017.00947)

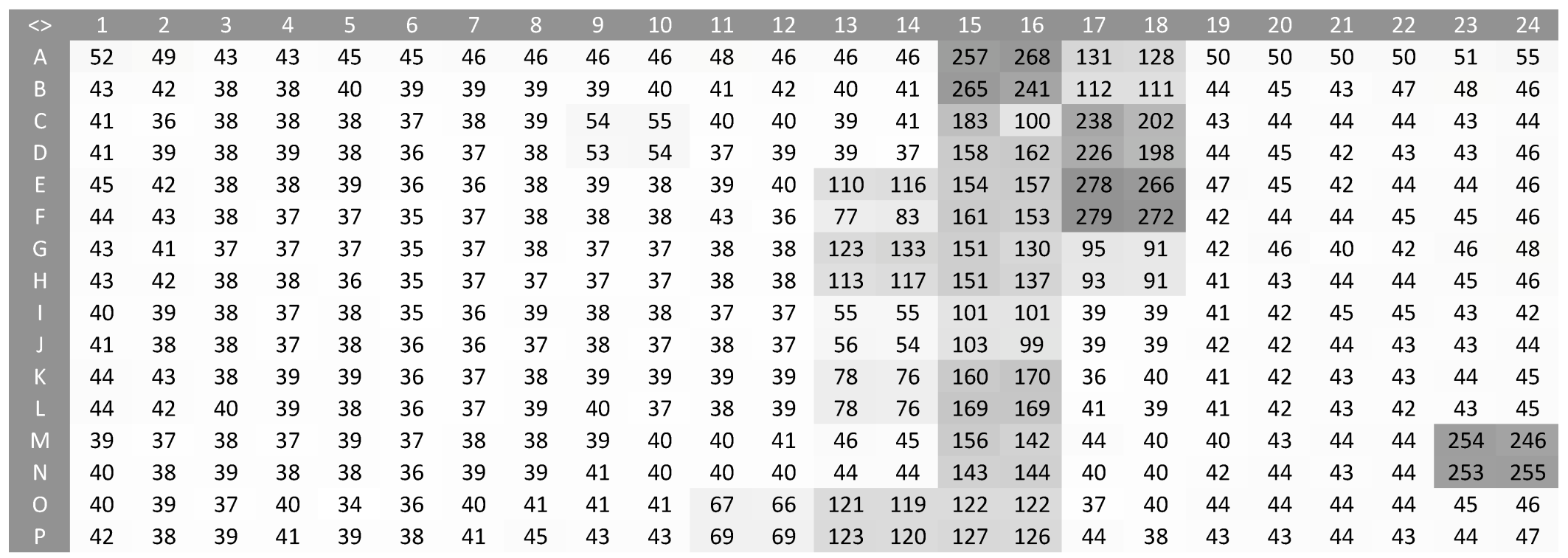

Supplement: FIGURE S3 — Fluorescence of compounds measured in a 384-well reference plate after 5 hours incubation with E. coli strain KMI001-AHK4 and addition of sodium carbonate solution. There were significant differences between fluorescence induced by several compounds and DMSO (in wells O23, O24, P23, P24), but these measured values did not affect the evaluation of the data set (the response induced by the internal standard was ∼6000 RFU). [file Image_3.TIF]

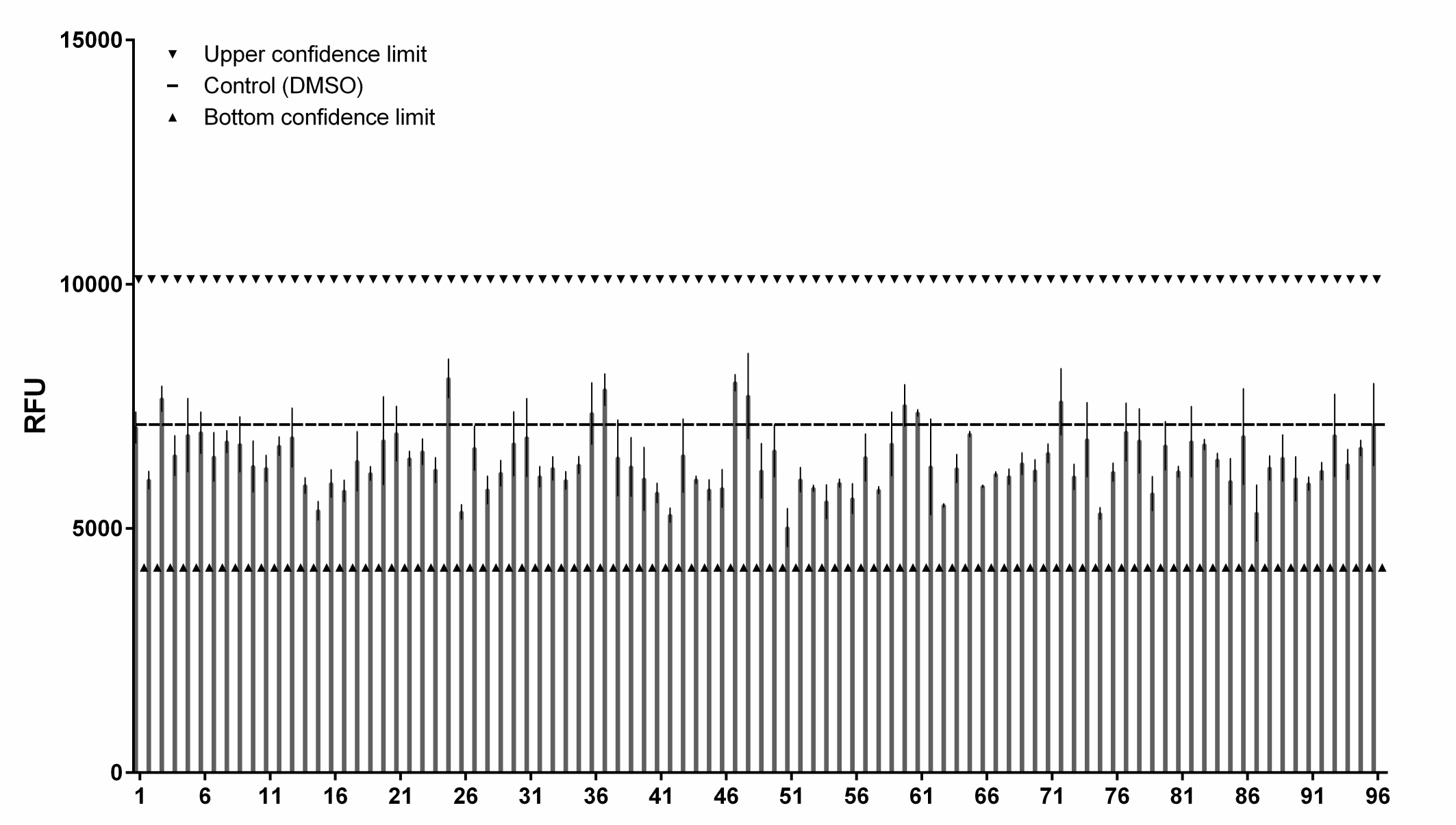

Supplement: FIGURE S4 — Quenching of fluorescence of compounds measured in a 384-well reference plate after 5 hours incubation with E. coli strain KMI001-AHK4 and addition of sodium carbonate solution and 4-MU. The measured fluorescence intensities were not significantly different from those induced by control (DMSO) (No. 96) at the significance level αADJ = 0.00054 according to Šidák correction. [file Image_4.TIF]
